# Molecular Modeling of Prion Transmission to Humans

**DOI:** 10.3390/v6103766

**Published:** 2014-10-02

**Authors:** Etienne Levavasseur, Nicolas Privat, Juan-Carlos Espinosa Martin, Steve Simoneau, Thierry Baron, Benoit Flan, Juan-Maria Torres, Stéphane Haïk

**Affiliations:** 1 Inserm U 1127, CNRS UMR 7225, Sorbonne Universités, UPMC Univ. Paris 06 UMR S 1127, Institut du Cerveau et de la Moelle épinière, ICM, 75013 Paris, France; E-Mails: etienne.levavasseur@inserm.fr (E.L.); nicolas.privat@inserm.fr (N.P.); 2 Centro de Investigacion en Sanidad Animal, Carretera de Algete a El Casar, 28130 Madrid, Spain; E-Mails: espinosa.juan@inia.es (J.-C.E.M.); jmtorres@inia.es (J.-M.T.); 3 LFB Biomédicaments, 91958 Les Ulis, France; E-Mails: simoneaus@lfb.fr (S.S.); flan@lfb.fr (B.F.); 4 Agence nationale de sécurité sanitaire de l’alimentation, de l’environnement et du travail (ANSES), Unité Maladies neurodégénératives, 69394 Lyon, France; E-Mail: thierry.baron@anses.fr; 5 AP-HP, Hôpital de la Pitié-Salpêtrière, Cellule nationale de référence des MCJ, F-75013 Paris, France; 6 AP-HP, Hôpital de la Pitié-Salpêtrière, Neuropathologie, 75013 Paris, France

**Keywords:** Prion, Protein misfolding cyclic amplification, molecular model, interspecies barrier

## Abstract

Using different prion strains, such as the variant Creutzfeldt-Jakob disease agent and the atypical bovine spongiform encephalopathy agents, and using transgenic mice expressing human or bovine prion protein, we assessed the reliability of protein misfolding cyclic amplification (PMCA) to model interspecies and genetic barriers to prion transmission. We compared our PMCA results with *in vivo* transmission data characterized by attack rates, *i.e.*, the percentage of inoculated mice that developed the disease. Using 19 seed/substrate combinations, we observed that a significant PMCA amplification was only obtained when the mouse line used as substrate is susceptible to the corresponding strain. Our results suggest that PMCA provides a useful tool to study genetic barriers to transmission and to study the zoonotic potential of emerging prion strains.

## 1. Introduction

Prion diseases are fatal transmissible disorders affecting humans and animals. They are characterized by brain vacuolization, neuronal loss and accumulation of PrPsc, an abnormal isoform of the host-encoded cellular prion protein (PrPc). PrPsc has been proposed as the infectious agent, capable of converting PrPc into PrPsc in an autocatalytical manner [1]. In humans, prion diseases result from contamination, genetic inheritance or sporadic event. The host susceptibility is influenced by the prion protein-encoding gene *PRNP*. For example, the variant of Creutzfeldt-Jakob disease (vCJD), which has been associated to the classical bovine spongiform encephalopathy (C-BSE) epidemics in cattle through contaminated meat product consumption [2], has occurred so far only in individuals homozygous for methionine at codon 129 of *PRNP* [3]. However, this finding has been under debate [4,5]. Studies have investigated interspecies and genotypic barriers [6,7] using an *in vitro* PrPsc amplification system named protein misfolding cyclic amplification (PMCA) [8]. This method allows, in PCR tubes, the amplification of minute amounts of PrPsc in infected tissues (seed) in the presence of normal brain homogenate in excess (substrate), after cycles of incubation and sonication. Then, the final product of the reaction can be detected after proteinase K digestion by Western blot. While PMCA allows the amplification of PrPsc, it has also been demonstrated that infectivity was increased during the reaction [9], and that prion strain properties were maintained throughout the reaction [10,11,12]. Brains from humans or transgenic mice expressing a human PrP with methionine at codon 129 of *PRNP* provided the best substrates to amplify vCJD and BSE PrPsc [6,7], suggesting that PMCA may reproduce faithfully the genotypic transmission barrier. It was thus proposed as a means to evaluate the zoonotic risk associated with emerging prion strains (Nor98 in sheep, L-type BSE in cattle). Indeed, while classical BSE strain has been recognized to be at the origin of vCJD in humans, L-BSE is considered to be a sporadic form of prion disease in cattle, differing in many aspects (epidemiology, neuropathology, biochemical features) from the C-BSE strain. Moreover, L-BSE has been transmitted more easily to transgenic mice overexpressing a human PrP [13,14] or to primates [15,16] than C-BSE. It has been suggested that some sporadic CJD subtypes in humans may result from an exposure to the L-BSE agent. Lending support to this hypothesis, pathological and biochemical similarities have been observed between L-BSE and an sCJD subtype (MV genotype at codon 129 of *PRNP*) [17], and between L-BSE infected non-human primate and another sCJD subtype (MM genotype) [15].

Therefore, we investigated whether PMCA is reliable to model the genotypic and interspecies barrier of transmission notably using L-BSE and C-BSE strains. We assessed the seeding ability of PrPsc from human (vCJD) and animal (C-BSE, L-BSE) prion strains to convert human or bovine PrPc. The aim of the study was to compare, for each seed/substrate combination, the result obtained by PMCA with the transmission efficiency previously observed *in vivo*.

## 2. Materials and Methods

### 2.1. Seeds and Substrates

The vCJD patient was referred to the French National Surveillance Network for CJD, and the diagnosis was confirmed neuropathologically. A written informed consent for autopsy and research use was provided by patient’s relatives, according to the French regulation (L.1232-1 to L.1232-3, Code de la Santé Publique). Brain homogenate used as seed for PMCA was prepared from frontal cortex.

Bovine brainstem tissues (C-BSE and L-BSE) were provided by the Agence nationale de sécurité sanitaire de l’alimentation, de l’environnement et du travail (ANSES, France). Mouse brains used as substrates for PMCA are recapitulated in Table 1.

**Table 1:** Description of the different mouse lines used in the present study. Brains from mice were used as substrates for Protein Misfolding Cyclic Amplification (PMCA).

Mouse Line	Reference	Transgene	Expression Rate (as Compared with)	Supplier
Tg650	[18]	HuPrPMet	x6 (human brain)	H. Laude, INRA, Jouy-en-Josas, France
Tg340	[19]	HuPrPMet	x4 (human brain)	J-M. Torres, CISA-INIA, Madrid, Spain
Tg152	[20]	HuPrPVal	x4–8 (human brain)	S. Prusiner, UCSF, San Francisco, USA
Tg362	[21]	HuPrPVal	x8 (human brain)	J-M. Torres, CISA-INIA, Madrid, Spain
Tg4092	[22]	BovPrP	x8–16 (bovine brain)	S. Prusiner, UCSF, San Francisco, USA
Tg110	[23]	BovPrP	x8 (bovine brain)	J-M. Torres, CISA-INIA, Madrid, Spain
RIII	[24]	-	endogenous MoPrP	F. Cortade, INRA, Nouzilly, France

[21] refers to unpublished data obtained by J.M. Torres. HuPrPMet: human PrP with a methionine at codon 129. HuPrPVal: human PrP with a valine at codon 129. BovPrP: bovine PrP. MoPrP: wild type mouse PrP.

### 2.2. Brain Homogenate Preparation

Animals were sacrificed in strict accordance with the ethical standards of French and European laws (European Community Council Directive 2010/63/EU of 22 September 2010).

Ten percent (wt/vol) normal brain homogenates (NBH) were prepared in conversion buffer (PBS with 150 mM NaCl, 1% Triton X-100, 0.005% EDTA pH 8, and Complete Protease Inhibitor Cocktail 1X (Roche)) using a FastPrep^®^ 24 instrument (MP Biomedical) for 45 s at speed 6.5, for use as fresh substrate (without a congelation step). Infected brain tissues were prepared similarly and stored at −80 °C, and used as seed for PMCA reaction.

### 2.3. Protein Misfolding Cyclic Amplification (PMCA)

To perform PMCA in conditions close to those of *in vivo* inoculation when using 10% crude brain homogenates of terminally ill subjects and to avoid the uncontrolled dilution of seed associated cofactors, we used only 10% crude homogenates prepared from human or bovine cases as seeds. Each seed/NBH mixture was prepared at 1:10 ratio and loaded into thin-walled 0.2-mL PCR tubes. A pre-PMCA aliquot (PMCA -) was immediately digested with PK 100 µg/mL for 1 hour at 37 °C. The remainder was incubated for 30 min at 37 °C and then subjected to 120 cycles of PMCA (one round), each cycle comprising a 30 s pulse at 75% potency (~200W) followed by an incubation of 7 min 30 s, using a Misonix S4000 sonicator (Misonix, Farmingdale, NY, USA). A post-PMCA aliquot (PMCA +) was then digested with PK as above and amplification of PrPsc was assessed by Western blot.

### 2.4. Western Blotting

Samples were electrophoresed on 4%–12% Bis-Tris NuPage gels in MES buffer (Life Technologies, St Aubin, France). Proteins were transferred to PVDF membrane using iBlot dry-transfer system (Life Technologies). Membranes were blocked (no blocking with mAb Sha31) for 15 min with 5% (wt/vol) milk powder in phosphate-buffered saline (PBS) containing 0.1% (vol/vol) Tween-20 and incubated for 1h with primary anti-PrP mAb, depending on the substrate (mAb 3F4, 1:10000 dilution, epitope 109–112 of human PrP sequence, Signet; mAb 6D11, 1:10000 dilution, epitope 93–109 of human PrP sequence, Covance; mAb Sha 31, 1:25000 dilution, epitope 145–152 of hamster PrP sequence, BertinPharma). It was followed by an incubation with secondary HRP-conjugated antibody (Pierce Biotechnology, Courtaboeuf, France) and chemiluminescent reaction was performed with ECL (GE Amersham, Velizy-Villacoublay, France). After exposure, a GS-800 densitometer and Quantity One software (Bio-Rad Laboratories, Marnes-la-Coquette, France) were used to quantify the PrPsc signal. An amplification factor (AF) was determined, as published by Jones *et al*. [7], and as follows: AF = (PMCA+/PMCA-). The “PMCA+” and “PMCA-” values were obtained by the densitometric analysis (optical density x area in mm^2^) of Western blot data from samples before (PMCA-) and after PMCA (PMCA+). When an important amplification was observed leading to a saturated signal in Western blot, the amplified product was serially diluted and the last quantifiable dilution was used to calculate the amplification factor, as previously described [25] and as follows: AF = (PMCA+/PMCA-) x dilution factor. The amplification factor was calculated as the mean value (±standard error of the mean) of at least three independent experiments for each substrate.

### 2.5. Statistical Analysis

Student’s *t* test (one-tailed) was used to assess whether the mean value of the amplification factor was statistically significantly greater than 1 (1 meaning no amplification) as described [25]. A Spearman correlation test and simple linear regression model were performed to study the relation between the amplification factor and survival times of animals. Analyses were performed using *Statview* [26] and *XLSSTAT* [27].

## 3. Results

**Figure 1:**
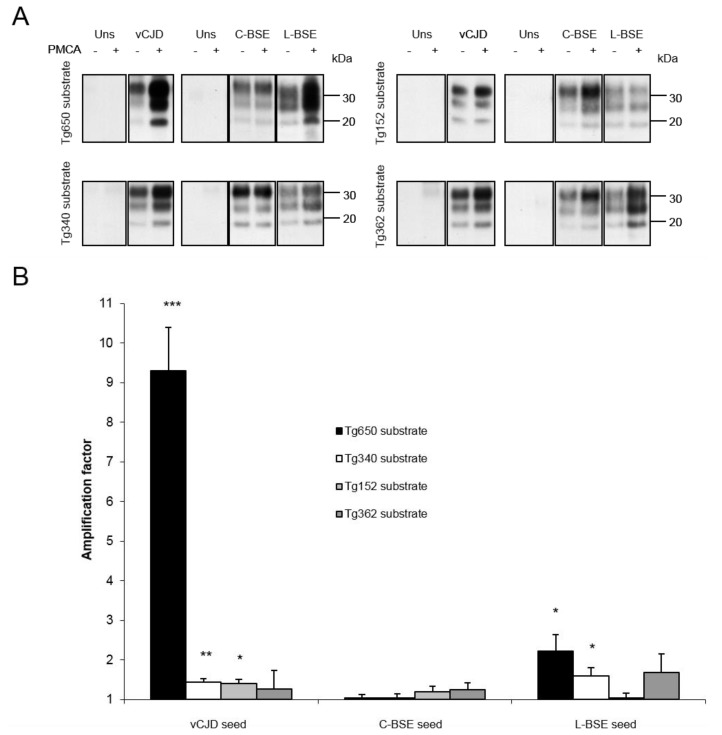
(**A**) Amplification of vCJD, classical BSE and L-type BSE PrPres by PMCA with substrates from mouse transgenic lines overexpressing human Met PrP (left panel) or Val PrP (right panel). Each blot is representative of at least 3 separate experiments. Blots were revealed using 3F4 mAb to detect vCJD PrPsc and Sha31 for BSE PrPsc; Uns: unseeded reaction. (**B**) Semiquantitative densitometric analysis of results obtained by Western blot. Error bars represent SEM. *** *p* = 0.001; ** p < 0.01; * p < 0.05*.*

All PMCA reactions were limited to one round of 120 cycles. Both transgenic mouse models overexpressing human PrP Met129 (tg650 and tg340) provided an efficient substrate for vCJD amplification (Figure 1). The highest amplification (AF > 9) was achieved with tg650 mice, while a lower amplification was obtained with tg340 mice. Variations were observed with brains from transgenic mice overexpressing human PrP Val129 (tg152 and tg362). Brain substrate from tg152 mice barely but significantly amplified vCJD PrPsc (Figure 1), while brains from tg362 mice showed no significant amplification in PMCA. With classical BSE, no significant amplification was detected when using brains from PrP-humanized mice as substrate (Figure 1), while L-BSE strain was readily amplified with brains from TgMet mice. With brains from TgVal mice, no amplification of L-BSE was observed with tg152 mice, and a slight but not significant amplification was obtained with tg362 mice (Figure 1).

**Figure 2:**
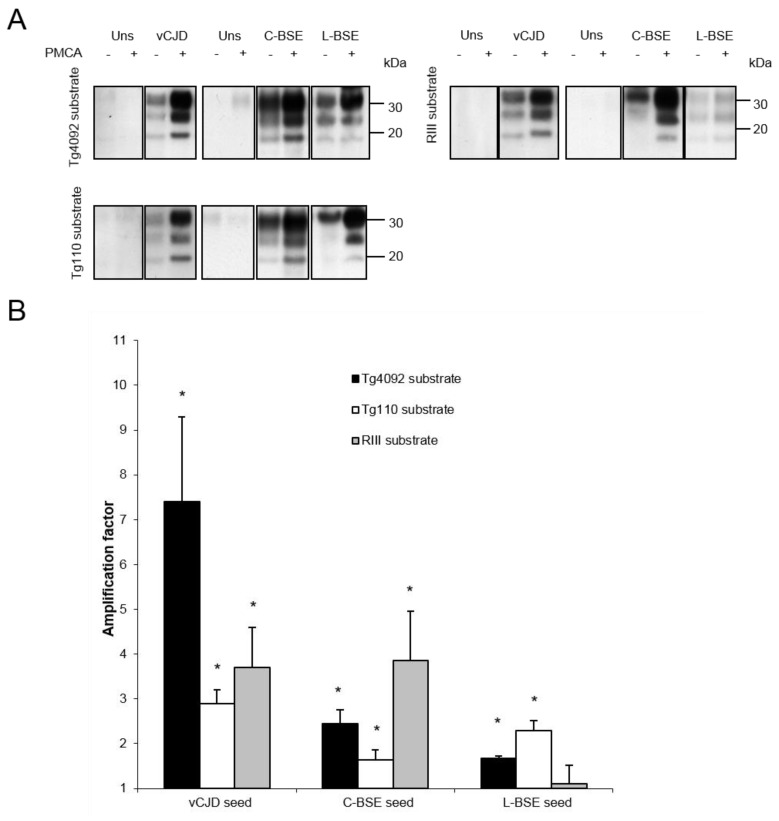
(**A**) Amplification of vCJD, classical BSE and L-type BSE PrPres by PMCA using substrates from 2 mouse transgenic lines overexpressing bovine PrP (left panel, revealed with Sha31 mAb) or from RIII mice (right panel, revealed with 6D11 mAb). Each blot is representative of at least 3 separate experiments; Uns: unseeded reaction. (**B**) Semiquantitative densitometric analysis of results obtained by Western blot. Error bars represent SEM. *** *p* = 0.001; ** p < 0.01; * p < 0.05.

With PrP-bovinized or RIII mice, the vCJD strain was efficiently amplified *in vitro* (Figure 2). Surprisingly, despite a PrP expression at a physiological level (*i.e.*, without PrP overexpression), brains from RIII mice were a substrate as efficient as brains from TgBov mice (Figure 2, Table 2). Likewise, the classical BSE strain was efficiently amplified with brain substrates from PrP-bovinized and RIII mice, and this latter model was even more efficient than TgBov mice (Figure 2, Table 2). On the contrary, brains from RIII mice did not provide an efficient substrate for the amplification of the atypical L-BSE strain, while an efficient amplification was obtained with substrates from both TgBov mouse lines (Figure 2).

**Table 2:** Comparison of the PMCA results with the known susceptibility *in vivo* of the different mouse lines used in the present study; [21] refers to unpublished data (0/6 mice developed the disease resulting in 0% attack rate; 6/6 mice developed the disease resulting in 100% attack rate); NA: not available; when attack rate was < 50% and amplification factor was not significantly different from 1, or when attack rate was > 50% and amplification factor was significantly different from 1, there was concordance (+) between *in vivo* susceptibility and *in vitro* amplification. In some cases, the comparison was not possible (“?”), due to lack of *in vivo* data.

Inoculum:	vCJD				C-BSE				L-BSE			
Mouse line:	Tg650	Tg340	Tg152	Tg362	Tg650	Tg340	Tg152	Tg362	Tg650	Tg340	Tg152	Tg362
Attack rate:	100%	100%	~50%	0%	<33%	<20%	<50%	0%	100%	100%	NA	0%
Reference:	[18]	[19]	[28]	[21]	[13]	[19]	[28]	[21]	[13]	[21]	NA	[21]
Amplification factor:	9.3	1.4	1.4	1.3	1	1	1.2	1.3	2.2	1.6	1	1.7
Concordance with PMCA results:	+	+	+	+	+	+	+	+	+	+	?	+
**Inoculum:**	**vCJD**			**C-BSE**			**L-BSE**		
Mouse line:	Tg4092	Tg110	RIII	Tg4092	Tg110	RIII	Tg4092	Tg110	RIII
Attack rate:	100%	100%	100%	100%	100%	100%	NA	100%	0%
Reference:	[29]	[23]	[30]	[22,29]	[23]	[30,31]	NA	[21]	[31]
Amplification factor:	7.4	2.9	3.7	2.5	1.6	3.9	1.7	2.3	1.1
Concordance witd PMCA results:	+	+	+	+	+	+	?	+	+

## 4. Discussion

For each strain/host combination, we compared PMCA results with *in vivo* data. When attack rate, defined as the proportion of inoculated animals that developed the disease, was below 50% and amplification factor was not significantly different from 1, or when attack rate was above 50% and amplification factor was significantly higher than 1, we concluded that there was concordance (+) between *in vivo* susceptibility and *in vitro* amplification. Our analysis was performed on 19 seed/substrate combinations. The aim of our study was not to make a quantitative comparison of the amplification efficiency obtained with the different substrates since differences in transgenic constructions and PrP expression levels (Table 1) or genetic background precluded such approach. We used the same PMCA parameters (incubation and sonication periods, power) for all the seed/substrate couples and only one round was performed, as published previously [7,25]. Our PMCA results and data from *in vivo* transmissions published by others or generated by the authors are recapitulated in Table 2.

Results obtained using PrP-humanized mouse brain substrates are consistent with the known susceptibilities *in vivo* of these animal models (Table 2). The highest amplification factor (>9) was obtained with the vCJD/ tg650 mice combination and is reminiscent of previous results obtained with a different knock-in mouse line expressing a human Met PrP [7]. However, such high efficiency obtained by us and others with some specific models [25] remains to be explained. While attack rates of tg650 and tg340 mice are identical (100%) when inoculated with the vCJD strain [18,19], the latter model was less efficient for vCJD PrPsc amplification, probably owing to the lower PrP expression in tg340 mice (Table 1). With substrates from tgVal mice, we observed a discrepancy between two models. The results obtained with brains from Tg362 mice showed no significant amplification in PMCA and were fully consistent with the 0% attack rate observed *in vivo* (Table 2). The low but significant amplification of the vCJD seed obtained with tg152 mice might reflect the average attack rate of ~50% observed with these mice inoculated with vCJD [28]. Importantly, this finding illustrates the possible variability between different mouse lines expressing the same PrP, and strongly suggests the need to include different mouse models in PMCA when assessing the species barrier for an emerging prion strain, in a similar manner to strain typing as it was originally performed.

The inefficient amplification of classical BSE as seed with brains from PrP-humanized mice as substrate suggests a significant interspecies barrier that is correlated with a low susceptibility in vivo (Table 2, [13,19,28]. Similarly, in agreement with the 100% attack rate obtained with *in vivo* transmissions (Table 2, [13]), brains from PrP-humanized mice (Met129) provided an efficient substrate for the amplification of L-BSE seed. This suggests a higher zoonotic potential associated with L-BSE than that associated with C-BSE, and is consistent with transmission data in non-human primates [15]. In this regard, PMCA may thus represent a useful tool for the risk assessment of such emerging strain.

Results obtained *in vitro* with substrates from mice expressing bovine or wild-type PrP were also in accordance with *in vivo* models, such as the vCJD strain that was efficiently amplified *in vitro*, matching results from *in vivo* transmissions [23,29,30]. Classical BSE seed was also readily amplified *in vitro* using TgBov and RIII mouse substrates, in accordance with *in vivo* experiments (100% attack rate) [22,23,29]. It is interesting to note that, for both vCJD and classical BSE seeds, RIII mice provided a very efficient substrate, despite the expression of a murine PrP at a physiological level. On the contrary, these mice did not provide an efficient substrate for the amplification of the L-BSE seed, and this was in agreement with known transmission data (Table 2). As expected with regard to the *in vivo* data (Table 2; [31,32]), brain substrate from TgBov mice allowed a significant amplification of L-BSE (Figure 2). Although brains from tg4092 mice provided an efficient substrate, the corresponding *in vivo* data are lacking, to our knowledge, for this model. However, we may infer from our PMCA results that an efficient *in vivo* transmission (with 100% attack rate) of L-BSE strain might be achieved in tg4092 mice.

Previous attempts to assess interspecies prion transmissibility using a cell-free conversion reaction have been made. Such assay is based on the conversion of purified radiolabeled PrPc to protease-resistant forms in presence of PrPres, and allows to investigate the role of the primary amino acid sequence of PrP from the seed and the substrate. Thus, the conversion efficiency of sheep PrP *in vitro* was correlated with the PrP polymorphism that governs sheep susceptibility to scrapie [33,34]. Likewise, a limited conversion efficiency of human PrP was obtained *in vitro* with BSE and scrapie PrPsc, in correlation with the low transmissibility of BSE to humans and the absence of evidence supporting the transmission of scrapie to humans. An efficient conversion of human PrP was observed with vCJD PrPsc [35]. However, it has also been found that such *in vitro* cell-free conversion system does not always reflect the biological behavior of prions *in vivo* [36]. This suggests that host factors distinct from PrP may modulate the conversion process and the transmission of prions, and by extent the species barrier. The PMCA assay, in which most molecular factors that could participate in the conversion event are present in the *in vitro* reaction, provides a complementary approach. To conclude, our PMCA results obtained with all the 19 seed/substrate combinations (with available *in vivo* data) were consistent with *in vivo* transmission data. Interestingly we obtained amplification factors in the same range (between 1 and 10) as those observed by other groups [7,25], suggesting a similar and robust PMCA efficiency despite setup variations between laboratories. Therefore, PMCA might represent a reliable method to evaluate the *in vivo* susceptibility of animals based on attack rate.
